# A prospective, randomized, single-blind, multicentre, phase III study on organ preservation with Custodiol-N solution compared with Custodiol® solution in organ transplantation (kidney, liver and pancreas)

**DOI:** 10.1186/s13063-019-3823-4

**Published:** 2020-01-10

**Authors:** Daniela Kniepeiss, Philipp Houben, Philipp Stiegler, Andrea Berghold, Regina Riedl, Judith Kahn, Peter Schemmer

**Affiliations:** 1 General, Visceral and Transplant Surgery, Department of Surgery, Medical University of Graz, Auenbruggerplatz 29, 8036 Graz, Austria; 2 Transplant Center Graz (TCG), Medical University of Graz, Auenbruggerplatz 29, 8036 Graz, Austria; 3 Department of General, Visceral and Transplant Surgery, University Hospital of Heidelberg, Heidelberg, Germany; 4 Institute for Medical Informatics, Statistics and Documentation, Medical University Graz, Graz, Austria

**Keywords:** Preservation solution, Liver transplantation, Kidney transplantation, Pancreatic transplantation

## Abstract

**Background:**

Organ preservation before transplantation is still a challenge. Both the University of Wisconsin and Bretschneider’s histidine-tryptophan-ketoglutarate (HTK; Custodiol®) solution are standard for liver, kidney and pancreas preservation. Organ preservation with both solutions is comparable; recently, however, Custodiol® solution has been modified to Custodiol-N according to the needs of today. Thus, our study was defined to study its effect in clinical transplantation.

**Methods:**

Patients undergoing kidney transplantation (*n* = 412) (including approximately 30 combined kidney–pancreas) or liver transplantation (*n* = 202) receive grafts that have been cold stored in either Custodiol® or Custodiol-N to demonstrate noninferiority of Custodiol-N regarding both graft function and graft injury after transplantation.

**Discussion:**

Preclinical data have clearly shown that Custodiol-N is superior to Custodiol® in cold static organ preservation via mechanisms including inhibition of hypoxic cell injury, cold-induced cell injury and avoidance of adverse effects during warm exposure to the solution. Further clinical safety data on Custodiol-N for cardioplegia are available. Thus, this study was designed to compare Custodiol® with Custodiol-N for the first time in a prospective, randomized, single-blinded, multicentre, phase III clinical transplantation trial.

**Trial registration:**

Eudra-CT, 2017–002198-20. Registered on 28 November 2018.

## Introduction

Bretschneider’s histidine-tryptophan-ketoglutarate (HTK; Custodiol®) solution was developed at the end of the 1970s and was first used in the setting of cardiac surgery as a cardioplegic solution in 1982. At the beginning of the 1980s, the solution began to be used for other purposes; first for kidney preservation, and later for liver and pancreas preservation as well. Since 2000, additional knowledge has been gained on the mechanisms of cell and tissue injury during cold ischemia. Based on these experimental findings, traditional Custodiol® solution has been modified to Custodiol-N (for the components of each, see Table 1).

**Table 1:** Components of Custodiol® and Custodiol-N

	Custodiol® (mmol/L)	Custodiol-N (mmol/L)
Sodium	16	16
Potassium	10	10
Magnesium	4	8
Calcium	0.015	0.020
Chloride	50	30
L-Histidine	198	124
N-α-acetyl-L-Histidine	–	57
Tryptophan	2	2
α-Ketoglutarate	2	2
Aspartate	1	5
Arginine	–	3
Alanine	–	5
Glycine	–	10
Mannitol	30	–
Sucrose	–	33
Deferoxamine	–	0.025
LK-614	–	0.0075

It has been shown in several cell types that the amino acids glycine and alanine provide protection against hypoxic or ischemic cell injury [1–7] which is attributed to a pathological membrane pore forming under hypoxic conditions and leading to alterations of the cellular ion homeostasis [3, 5, 8]. Furthermore, it has become clear that hypothermia, used to protect tissues against ischemic injury, also triggers injury on its own account [9–13]. This cold-induced cell injury affects numerous cell types but, in particular, affects endothelial cells, jeopardizing vascular function after reperfusion, and is mediated by intracellular “redox-active” iron [11, 14–16]. Based on these experimental findings, the traditional Custodiol® solution was fortified with the amino acids glycine and alanine to inhibit the formation of the hypoxia-induced plasma membrane pore. Furthermore, it was supplemented by the strong, but poorly membrane-permeable, iron chelator deferoxamine and the new, membrane-permeable iron chelator LK-614 to inhibit cold-induced cell injury.

Furthermore, recent studies have shown that the buffer histidine, essential for the efficiency of Custodiol® (see above), can have adverse effects on some cell types rich in “redox-active” iron, particularly if used at very high concentrations [17]. The histidine derivative N-acetyl-histidine does not display these adverse effects but has a similar buffering power to histidine. Therefore, part of the histidine in Custodiol® (as much as possible without altering the successful ion composition of the solution) was replaced by the superior derivative N-acetyl-histidine.

Microcirculatory disturbances have been reported following reperfusion of diverse organs. In various experimental models, the troublesome application of the vasodilator nitric oxide or simply the supplementation of L-arginine (the substrate of the endogenous nitric oxide-producing enzymes) has been proven to decrease these microcirculatory disturbances [18–21]. Therefore, in Custodiol-N, L-arginine is supplemented. As mannitol is not impermeable to all cell types (i.e. hepatocytes [22]), it has been replaced with saccharose. Although efficient buffering is important to prevent severe acidosis, moderate acidosis has been shown to provide protection against ischemic injury [23, 24]; therefore Custodiol-N has a slightly lower pH than Custodiol®. Finally, aspartate has been added to replenish intermediates of the tricarboxylic acid cycle in combination with the ketoglutarate that is already present in Custodiol® for an efficient energy production after reperfusion. Besides these alterations, the successful composition of Custodiol® has been maintained.

This amino acid-fortified and iron chelator-supplemented Custodiol-N proved to be superior to Custodiol® in in vitro studies using diverse cell types; in particular, Custodiol-N showed far superior inhibition of hypoxic cell injury, superior to far superior inhibition of cold-induced cell injury, and avoidance of adverse effects; this was also seen during warm exposure to the solution [14, 25].

In a study on the isolated perfused rat heart (Langendorff model), a protective effect towards the injury to the coronary vasculature inflicted by cold ischemia was shown by the iron chelators deferoxamine and LK-614, that is the iron chelators present in Custodiol-N [26].

A further study demonstrated that Custodiol-N protects liver grafts with microvesicular steatosis caused by acute toxic injury from cold ischemic injury better than Custodiol®, most likely via inhibition of hypoxic injury and oxidative stress and amelioration of the inflammatory reaction occurring upon reperfusion [27].

## Objectives

The objective of this clinical trial is to demonstrate noninferiority of graft preservation with Custodiol-N compared to Custodiol® with respect to both graft function and injury after transplantation of kidney, liver or combined kidney–pancreas.

### Primary end points

#### Kidney

The primary end point after kidney transplantation is delayed graft function, defined as dialysis requirement during the first week after transplantation.

#### Liver

After liver transplantation, the primary end point is the area under the curve (AUC) of alanine aminotransferase (ALT) during the first 7 days after surgery (minimum one measurement per day).

### Secondary objectives

#### Kidney

The secondary objectives after kidney transplantation are the incidence of primary poor function defined as creatinine >250 μmol/l after 90 days, serum creatinine, creatinine modification of diet in renal disease (MDRD) clearance, urea and haemoglobin after 90 days, and the requirement for dialysis until 90 days after transplantation, as well as biopsy-proven rejection.

#### Combined kidney–pancreas

The secondary end points after pancreatic transplantation include insulin requirements on postoperative days 3, 30 and 90. Furthermore, they include α-amylase, lipase, and C-reactive protein levels at 1 and 3 days, and both C-peptide and glycated haemoglobin levels at postoperative days 30 and 90. Moreover, pancreatic complications (i.e. graft pancreatitis, anastomotic leak and vascular complications including thrombosis, stenosis, and bleeding) will be compared, while fasting glucose after 90 days will be documented.

#### Liver

The secondary end points after liver transplantation include the absolute peak serum lactate dehydrogenase (LDH) level within the first 7 days after transplantation, the peak serum ALT and LDH levels, and serum bilirubin, aspartate aminotransferase (AST), ALT, LDH, and total albumin levels, and prothrombin time at postoperative day 90. Further biliary complications (i.e. the number of episodes of cholestasis, therapy for cholangitis, episodes of biliary leakage and intrahepatic and/or extrahepatic biliary strictures) will be assessed.

## Methods/design

This multicentre study is designed as a prospective, randomized and single-blinded trial. It is a phase III comparison study of organ preservation intended to demonstrate the noninferiority of Custodiol-N compared with Custodiol® in organ transplantation for the kidney, liver and pancreas. Study centres are the General, Visceral and Transplant Surgery, Department of Surgery, Medical University of Graz, Austria, and the Visceral, Transplant and Thoracic Surgery, Department of Surgery, Medical University of Innsbruck, Austria (Additional file 1).

### Inclusion criteria

#### Donor (liver, kidney, and combined kidney–pancreas) criteria

Deceased adult donors (≥18 years) fulfilling the criteria for organ donation are included in the study.

#### Patient (recipient) criteria

Patients (≥18 years) eligible for whole organ transplantation are included in the study.

### Exclusion criteria

Generally, patients with previous liver, kidney and combined kidney–pancreas transplantation are excluded.

#### Donor (liver, kidney, and combined kidney–pancreas) criteria

Donors whose organs are allocated to be used outside of Austria, a general refusal of organ donation, and being a donor after death due to cardiac causes are excluded.

#### Patient (recipient) criteria

Pregnant or lactating patients, recipients participating in any other interventional study (e.g. studies involving compounds/interventions aimed at the reduction of preservation and/or ischemia/reperfusion injury) and all combined allocations other than the pancreas and kidney are excluded from the study.

#### Kidney recipients

Double-kidney transplant patients, those on machine perfusion, and all patients with panel reactive antibody >0% according to the Kidney Disease Improving Global Outcomes 2009 guidelines are excluded.

#### Liver recipients

Machine-perfused organs are excluded from the study as well as recipients undergoing re-transplantation.

### Sample size

The study will continue until 412 kidney recipients and 202 liver recipients have been enrolled, treated and followed up. The number of combined kidney–pancreas transplants will be purely incidental and is estimated to be around 30. The overall duration for the trial is expected to be approximately 48 months. The duration of the trial for each participant is expected to be 3 months (for transplantation and a follow-up period of 90 days).

### Withdrawal of patients

Patients who discontinue participation in the clinical study on their own will be defined as premature withdrawals. Every patient has the right to withdraw their consent to participation in the clinical trial at any time and without giving reasons. This will not cause them to be disadvantaged. Nevertheless, the investigator must make every effort to find out the reasons why the patient has withdrawn from the study, while taking care not to compromise the patient’s rights. The time of withdrawal, the results available up to that time and, if known, the reason for the patient’s withdrawal are to be documented in the trial report form.

All ongoing adverse events (AEs)/serious adverse events (SAEs) in withdrawn participants have to be followed up until no more signs and symptoms are verifiable or the participant is in a stable condition. Patients who withdraw will not be replaced.

### Study medication

#### Treatment assignment

The reported organ donors are allocated according to the Eurotransplant guidelines without influence from the study. Only organs allocated to a patient who has signed the informed consent will be randomized.

Multiorgan donation is performed according to institutional guidelines. Organ perfusion is performed using Custodiol® as the standard preservation solution. Subsequently, organs allocated to a study centre with patients who have signed the informed consent will be randomized to either study medication. In case of simultaneous transplantation of the kidney and pancreas, both organs will be randomized together for the same study arm.

After randomization, back table perfusion with study medications will be performed via the renal artery for the kidney, the hepatic artery as well as the portal vein for the liver, and the mesenteric artery and portal vein for the pancreas using approximately 250–1000 ml of study medication per organ (Custodiol® or Custodiol-N). Subsequently, the grafts will be stored in 1000 ml of the study medication. The allocated study medication is provided to the recipient site. Before transplantation, back table perfusion using approximately 250–1000 ml of the study medication will be optionally performed.

#### Randomization and blinding

Recipients with signed informed consent meeting all inclusion criteria and without any exclusion criteria will undergo transplantation with grafts that have been randomized to Custodiol® or Custodiol-N in a blinded manner. Grafts are randomized in a 1:1 ratio to receive either Custodiol® or Custodiol-N. After randomization, the randomized solution will be prepared for treatment at the donor site. Study medication will not be blinded for the investigator. Randomization will be performed using the web-based randomization service “Randomizer for Clinical Trials” developed at the Institute for Medical Informatics, Statistics and Documentation, Medical University of Graz, Austria.

#### Packaging and labelling

Trial medication will be provided by the sponsor as 1-litre bags (3 litres for one patient) and the appropriate amount of lyophilisate per bag to the distributing pharmacy. Packages will be opaque and labelled with either “Custodiol®” or “Custodiol-N” in an unblinded fashion and with the study title, EudraCT number and “Zur klinischen Prüfung bestimmt”, according to the Directive 2001/20/EC of the European Parliament. Each bag will be labelled in the same way. To ensure allocation concealment, clinical monitors will check that unused medication packages have not been opened.

### Study procedure

**Fig. 1:**
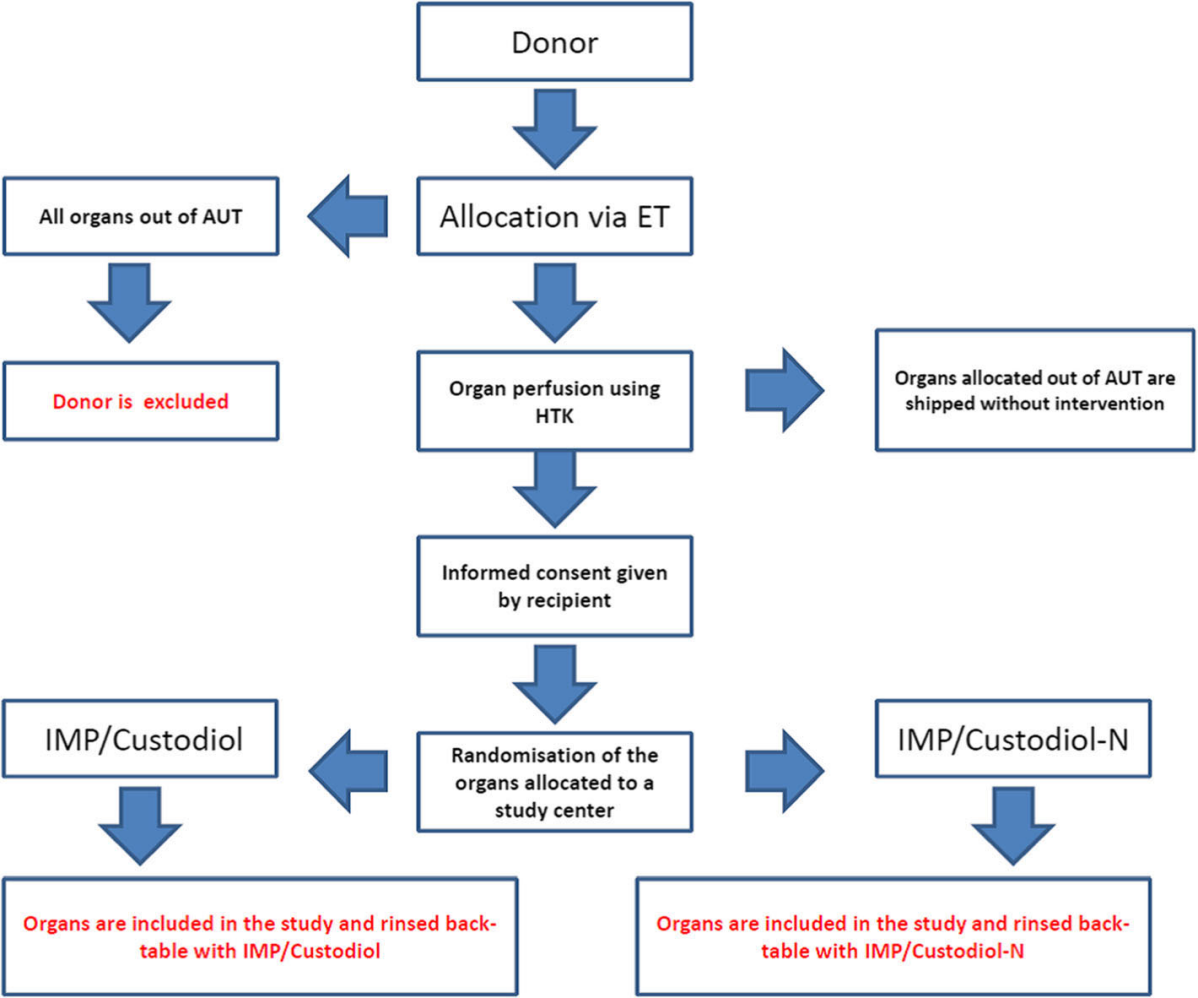
Consolidated Standards of Reporting Trials (CONSORT) flowchart of randomization. AUT Austria, ET Eurotransplant, HTK histidine-tryptophan-ketoglutarate, MELD investigational medicine product

The trial will begin when history taking has shown that the criteria for inclusion in the trial have been met (performed by the study team of transplant surgeons), and when the patient has been fully informed regarding the objective of the study, the trial product, the risks and the insurance cover, and when the signed declaration of consent is available. Allocation principles are shown in Fig. 1.

#### Organ perfusion technique

The perfusion of all abdominal organs with cold (2–8°C) Custodiol® will be performed according to the standard procedure. Briefly, after mobilization and appropriate preparation of intra-abdominal organs and blood vessels and clamping of the aorta above the superior mesenteric artery, perfusion with Custodiol® is started via the infrarenal aorta. The duration of perfusion is usually 10–15 min. During perfusion, the abdominal cavity is cooled with cold physiological NaCl solution. Cold storage of grafts must be performed in the solution used for perfusion of the donor.

### Kidney

#### Screening of donor and graft status

**Fig. 2:**
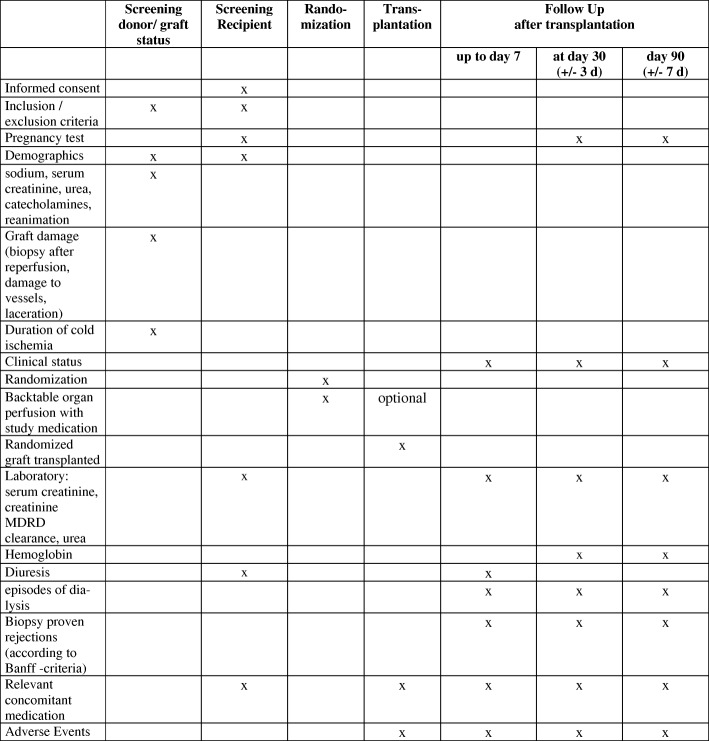
Template of content for the schedule of enrolment, interventions and assessments. SPIRIT figure for kidney transplantation. MDRD modification of diet in renal disease

The screening visit includes a check of the inclusion and exclusion criteria, assessment of demographic data (age and gender), the clinical status (sodium, serum creatinine, urea, catecholamines (yes/no)), mechanical or medicamentous cardiopulmonary resuscitation of the donor (yes/no), the graft damage (biopsy after reperfusion, damage to vessels, laceration), and duration of cold ischemia (Fig. 2).

#### Screening of the recipient

The screening visit includes a check of the inclusion and exclusion criteria (to test female patients ≤55 years for pregnancy), assessment of demographic data (age and gender) of the recipient, and informed consent by the study team. Within 24 h before transplantation, the recipients’ routine laboratory results (serum creatinine, creatinine MDRD clearance and urea) are recorded. Diuresis is also assessed. Additionally, the occurrence of adverse advents and concomitant medication will be recorded. Events compromising the blinding of the patient will be recorded as protocol deviations.

#### Follow-up

Follow-up will be performed daily up to day 7, and at postoperative days 30 (±3 days) and 90 (±7 days). These follow-up visits assess the recipient’s clinical status (American Society of Anesthesiologists (ASA) classification), episodes of dialysis, the recipients’ routine laboratory results (serum creatinine, creatinine MDRD clearance, urea) and the diuresis per hour (up to 7 days). Haemoglobin will be analysed on day 30 and after 90 days. Additionally, the occurrence of biopsy-proven rejections (according to Banff criteria) and adverse advents are recorded. Relevant concomitant medication (catecholamines, immunosuppression, anti-infectives, antihypertensives, antiarrhythmics, and diuretics) will be recorded in detail up to day 7 of follow-up and at days 30 and 90 after transplantation as ‘yes’ or ‘no’ only.

### Liver

#### Screening of donor and graft status

**Fig. 3:**
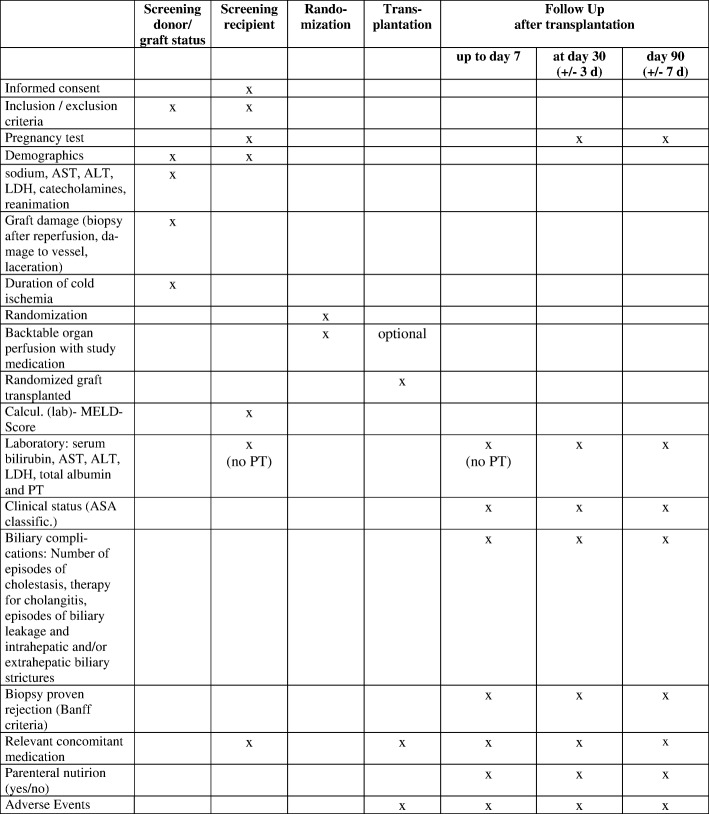
Template of content for the schedule of enrolment, interventions and assessments. SPIRIT figure for liver transplantation. ALT alanine aminotransferase, ASA American Society of Anesthesiologists, AST Aspartate aminotransferase, LDH lactate dehydrogenase, MELD model for end-stage liver disease, PT prothrombin time

The screening visit includes a check of the inclusion and exclusion criteria, assessment of demographic data (age and gender) and the clinical status (sodium, AST, ALT, LDH, bilirubin, catecholamines (yes/no), reanimation (yes/no)) of the donor, the graft damage (biopsy after reperfusion, damage to vessels, laceration), biopsy (steatosis (micro-/macrovesicular)), inflammatory changes, fibrosis and duration of cold ischemia (Fig. 3).

#### Screening of the recipient

The screening visit includes a check of the inclusion and exclusion criteria (to test female patients ≤55 years for pregnancy), assessment of demographic data (age and gender) of the recipient, and informed consent. Within 24 h before transplantation, the recipients’ clinical status (calculated model for end-stage liver disease score) and the recipients’ routine laboratory results (serum bilirubin, AST, ALT, LDH, total albumin) are assessed. Additionally, the occurrence of adverse advents and concomitant medication will be recorded. Events compromising the blinding of the patient will be recorded as protocol deviations.

#### Follow-up

Follow-up will be performed daily up to day 7, and at postoperative days 30 (±3 days) and 90 (±7 days). These follow-up visits assess the recipient’s clinical status (ASA classification), biopsy-proven rejection episodes (according to Banff criteria), the recipient’s routine laboratory results (serum bilirubin, AST, ALT (a minimum of two times per day between days 1–7), LDH, total albumin) and prothrombin time after 30 days and 3 months, and biliary complications (number of episodes of cholestasis, therapy for cholangitis, episodes of biliary leakage and intrahepatic and/or extrahepatic biliary strictures). Additionally, the occurrence of any adverse advents will be recorded. Relevant concomitant medication (catecholamines, immunosuppression, anti-infectives, antihypertensives, antiarrhythmics, and diuretics) will be recorded in detail up to day 7 of follow-up and at days 30 and 90 after transplantation as ‘yes’ or ‘no’ only. Graft survival will be recorded, and parenteral nutrition will be checked.

### Combined kidney–pancreas

#### Screening of donor and graft status

**Fig. 4:**
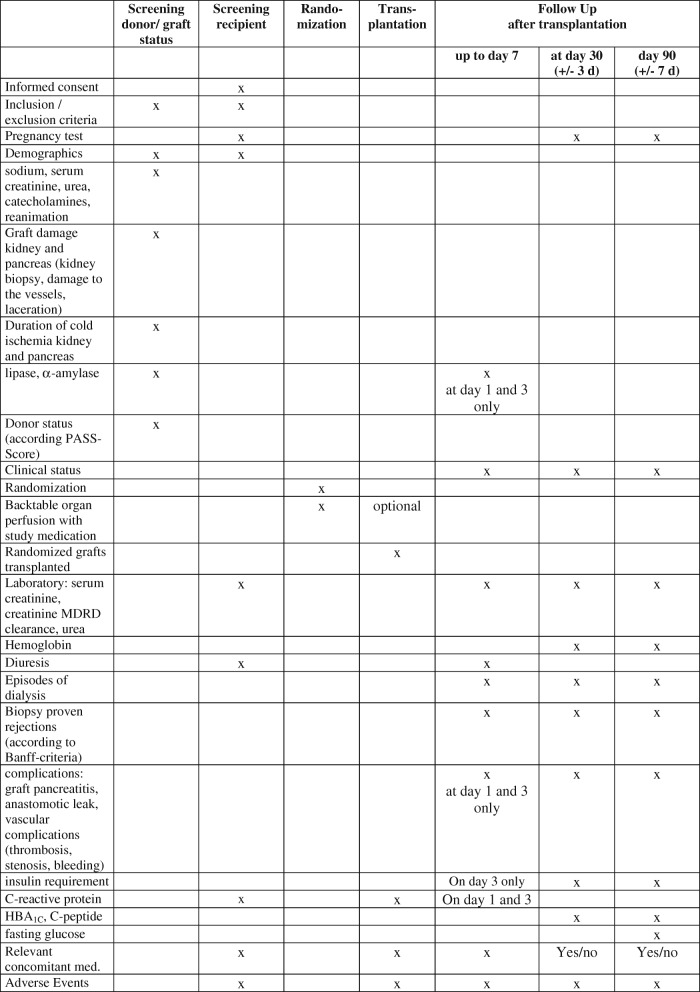
Template of content for the schedule of enrolment, interventions and assessments. SPIRIT figure for pancreatic transplantation. MDRD modification of diet in renal disease, PASS Pancreas Allocation Suitability Score

The screening visit includes a check of the inclusion and exclusion criteria, assessment of demographic data (age and sex), the clinical status (sodium, serum creatinine, urea, catecholamines (yes/no), reanimation (yes/no) of the donor) and the graft damage (kidney biopsy, damage to vessels, laceration). Additionally, the donor status according to the Pancreas Allocation Suitability Score and the duration of cold ischemia for the pancreas and kidney are recorded (Fig. 4).

#### Screening of the recipient

The screening of the recipient includes a check of the inclusion and exclusion criteria (to test female patients ≤55 years for pregnancy), assessment of demographic data (age and gender) of the recipient, and informed consent. Within 24 h before transplantation, in addition to the recipient’s clinical status (ASA classification) and routine laboratory results (serum creatinine, creatinine MDRD clearance, urea) the recipient’s C-reactive protein is recorded. Diuresis is also assessed.

#### Follow-up

Follow-up will be performed daily up to day 7, and at postoperative days 30 (±3 days) and 90 (±7 days). These follow-up visits assess the recipient’s clinical status (ASA classification), episodes of dialysis, the recipient’s routine laboratory results (serum creatinine, creatinine MDRD clearance, urea) and the diuresis per hour (up to 7 days). Haemoglobin will be analysed on day 30 and after 90 days. Additionally, the occurrence of biopsy-proven rejections (according to Banff criteria) and adverse advents are recorded. Relevant concomitant medication (catecholamines, immunosuppressors, anti-infectives, antihypertensives, antiarrhythmics, diuretics) will be recorded in detail up to day 7 of follow-up and at days 30 and 90 after transplantation as ‘yes’ or ‘no’ only. Follow-up visits at day 1, day 3, day 30 and day 90 will assess pancreatic complications such as graft pancreatitis and anastomotic leak and vascular complications (thrombosis, stenosis, bleeding). The routine laboratory findings (lipase, α-amylase and C-reactive protein) are measured on day 1 and 3 days after transplantation. Further insulin requirements on days 3 and 30 (±3 days) and after 90 days will be assessed. The glycated haemoglobin level and C-peptide level are measured at days 30 and 90 after transplantation. Fasting glucose will also be documented 90 days after transplantation. Adverse events are documented up to day 90. Pancreas-relevant concomitant medication (insulin/antidiabetics) will be documented in detail up to day 3 of follow-up and at days 30 and 90 after transplantation as ‘yes’ or ‘no’ only. Graft survival will be recorded.

### Data management

#### Data collection and handling

All information required by the protocol and collected during the trial must be entered by the investigator, or a designated representative, in the electronic case report form (eCRF). Each patient will be assigned a site number and a site-specific screening number reflecting the chronological order of screening. Any link of the site number to a patient’s identity will be kept confidential at the study site. Each donor organ will be identified by a randomization code while the type of organ is conveyed implicitly; right and left kidneys will be identified. The investigator, or a designated representative, should complete the eCRF pages as soon as possible after the information is collected, preferably on the same day as a trial participant is seen for an examination, treatment, or any other trial procedure. Any pending entries must be completed immediately after the final examination. Explanation should be given for all missing data.

Data management is accomplished according to the appropriate standard operating procedures valid in the Institute for Medical Informatics, Statistics and Documentation, Medical University Graz, Austria.

After completion of the query process, the database will be closed and exported into the statistical evaluation system for analysis.

#### Storage and archiving of data

All important trial documents (e.g. the eCRF) will be archived by the sponsor for at least 10 years after the trial termination.

The investigator(s) will archive all trial data (source data and the Investigator Site File including the participant identification list and relevant correspondence) according to section 4.9 of the International Council for Harmonisation (ICH) Consolidated Guidelines on Good Clinical Practice (GCP) (E6) and according to local law or regulations [28].

### Statistical analysis

#### Sample size determination

Sample sizes have been calculated for the kidney and liver. The trial will stop once both sample sizes have been reached.

Very few patients are expected to be lost to follow-up as they will be under close clinical observation. Also, a patient’s active termination from the study is expected to happen very rarely as very few procedures will be study-specific and personal data will be used for transplantation registers anyway. Therefore, no additional patients are planned to be enrolled to make up for attrition effects.

#### Kidney

In a systematic review by O’Callaghan et al. [29], delayed graft function was chosen as the main end point. For the largest trials assessing HTK solutions, the rates for this vary at around 30%. For Custodiol-N, an increase of this rate by one-half to 45% has been defined as noncritical. In order to reject the hypothesis that the delayed graft function rate increases by 15% or more at the alpha level of 0.025 with a power of 90%, 412 patients are needed. This sample size calculation is based on the score test of Farrington and Manning for a noninferiority test comparing two proportions. Autocorrelation effects caused by the transplant of two kidneys per donor to different patients have been assessed as negligible.

#### Liver

The success of liver transplantation is determined by the AUC of ALT measurements over the first 7 days after implantation. In a sample of 25 consecutively treated patients, the ALT level in the period between surgery and 5 days had an AUC of about 3800 mg*d/dl with a standard deviation of about 2000 mg*d/dL. Assuming a coefficient of variation of 0.5, in order to reject the hypothesis that the peak level rises by 25% or more at the alpha level of 0.025 with a power of 90%, 202 patients are needed. For sample size calculation, a two-sample *t* test of the mean ratio with lognormal data was used.

#### Combined kidney–pancreas

Very few pancreata are transplanted in Austria. We do not expect to collect enough data in a reasonable amount of time about the success rates of pancreatic transplantation. Therefore, data on success and safety will be collected on these patients but the results will only be presented in a descriptive way.

#### Analysis sets

All organs randomized and implanted or with an attempt to implant will be included in the analysis according to the randomized treatment group. Organs randomized but not implanted will count as screening failures.

All patients treated according to the study protocol will be included in the per-protocol set. A list of protocol deviations sufficient to exclude patients from this set will be compiled by the sponsor, the main investigator and the biostatistician before analysis starts. The primary analysis will be performed on the per-protocol set.

All tests of the primary criteria with respect to the liver and kidney would have to successfully reject the null hypothesis to support a favourable result with respect to Custodiol-N. Null hypothesis tests will be repeated for intention-to-treat sets.

### Harms

#### Adverse events

According to GCP, an AE is defined as follows: any untoward medical occurrence in a participant administered a pharmaceutical product and which does not necessarily have a causal relationship with this treatment. An AE can therefore be any unfavourable and unintended sign (including an abnormal laboratory finding), symptom, or disease temporally associated with the use of a medicinal (investigational) product, whether or not related to the medicinal (investigational) product.

An AE may be a new symptom/medical condition, new diagnosis, change in laboratory parameters, intercurrent disease or accidents, worsening of medical conditions/diseases existing before the start of the clinical trial or recurrence of disease.

A pre-existing disease or symptom will not be considered an adverse event unless there is an untoward change in its intensity, frequency or quality. This change will be documented by an investigator.

Surgical procedures themselves are not AEs; they are therapeutic measures for conditions that require surgery. The condition for which the surgery is required may be an AE. Planned/elective surgical measures and the condition(s) leading to these measures are not AEs if the condition leading to the measure was present prior to the inclusion into the trial. All AEs (inclusive of SAEs) will be documented on an AE form. AEs are classified as “nonserious” or “serious”.

#### Serious adverse event

An SAE is one that at any dose (and also overdose) results in death, is life-threatening (the term life-threatening refers to an event in which the participant was at risk of death at the time of event and not to an event which hypothetically might have caused death if it was more severe), requires hospitalization of the participant or prolongation of existing hospitalization, results in persistent or significant disability/incapacity*,* is a congenital anomaly/birth defect or is otherwise medically relevant. All SAEs will additionally be documented on an SAE form.

#### Expectedness

An ‘unexpected’ adverse event is one in which the nature or severity is not consistent with the applicable product information, e.g. the Investigator’s Brochure. Furthermore, reports which add significant information on specificity or severity of a known adverse reaction constitute ‘unexpected’ events. Specific examples would be acute renal failure as an expected adverse reaction with a subsequent new occurrence of interstitial nephritis and hepatitis with a first occurrence of fulminant hepatitis.

#### SUSAR

SAEs that are both suspected, i.e. possibly related to the medicinal investigational product, and ‘unexpected’, i.e. the nature and/or severity of which is not consistent with the applicable product information (Investigator’s Brochure), are to be classified as suspected unexpected serious adverse reactions (SUSARs).

In cases where either the investigator who primarily reported the SAE or the second assessor classifies the SAE as ‘suspected’, i.e. related to the medicinal investigational product, and the SAE is ‘unexpected’, it will be categorized as a SUSAR. All SUSARs are subject to expedited reporting to the responsible ethics committee(s), the competent higher federal authority (“Bundesamt für Sicherheit im Gesundheitswesen”) and to all participating investigators.

#### Period of observation and documentation

AEs will be documented from the time of transplantation up to the last follow-up visit at day 90. All participants who present AEs, whether considered associated with the use of the trial medication or not, will be monitored by the responsible investigator to determine their outcome. The clinical course of the AE will be followed up until resolution/normalization of the changed parameter or until achievement of a stable condition.

### Ethics

#### Good clinical practice

The procedures set out in this trial protocol pertaining to the conduct, evaluation, and documentation of this trial are designed to ensure that all persons involved in the trial abide by GCP and the ethical principles described in the applicable version of the Declaration of Helsinki. The trial will be carried out in keeping with local legal and regulatory requirements.

#### Patient information and informed consent

Before being admitted to the clinical trial, a potential participant must consent after the main features of the clinical trial have been explained in a form understandable to them. Consent has to be given after patient education about transplantation and before the transplantation procedure. The participant must give consent in writing. The signed informed consent form will be filled in by the study team. A copy of the signed informed consent document must be given to the participant. The documents must be in a language understandable to the participant and must specify who informed the participant about the trial. The participants will be informed as soon as possible if new information may influence their decision to participate in the trial. The communication of this information should be documented.

#### Confidentiality

The data obtained during the course of this trial will be treated pursuant to the Austrian Datenschutzgesetz 2000.

During the clinical trial, participants will be identified solely by their year of birth and an individual identification code (participant number, randomization number). Trial findings stored on a computer will be stored in accordance with local data protection law and will be handled in the strictest confidence. For the protection of these data, organizational procedures are implemented to prevent distribution of data to unauthorized persons. The appropriate regulations pertaining to local data legislation will be fulfilled in their entirety.

The participant consents in writing to release the investigator from their professional discretion in so far as to allow inspection of the original data for monitoring purposes by health authorities and authorized persons (inspectors, monitors, auditors). Authorized persons (clinical monitors, auditors, inspectors) may inspect the participant-related data collected during the trial ensuring the data protection law. The investigator will maintain a participant identification list (participant numbers with the corresponding participant names) to enable records to be identified.

Participants who did not consent to circulate their pseudonymized data will not be included into the trial.

### Quality assurance

#### Monitoring

The study will be performed in accordance with Good Clinical Practice and will thus require regular monitoring visits. Monitoring will be done by personal visits from a clinical monitor according to standard operating procedures. Monitoring visits will occur based on patient accruals and the availability of eCRFs. The monitor will review the entries into the eCRFs on the basis of source documents. Details of monitoring (i.e. frequency of visits and/or the extent of source data verification) will be specified in the monitoring manual for this trial. Between these visits, contacts with study site personnel will be made by telephone, by fax or by mail, to ensure that the trial is conducted according to the protocol and the regulatory requirements.

#### Source documents

For each patient included in the study, a specific file (i.e. institution file) must exist containing original data, on which the information recorded on the eCRF is based. Source documents and eCRFs must not be exact copies of each other. As a general rule, medical information that is not specifically required by the study (e.g. patient gender, prior medical history, prior medication, type of surgical procedure, and so forth) must be found in the source medical documents (and on the eCRF). Information specifically required by the protocol and not required by routine clinical care may be recorded directly onto the eCRF without appearing in the source documents. In addition, source documents must mention that the patient has been included in an investigational study. Finally, there must be no data that are inconsistent between the eCRF and source documents.

### Dissemination

All important trial documents (e.g. the eCRF) will be archived by the sponsor for at least 15 years after the trial termination. The investigator(s) will archive all trial data according to the section 4.9 of the ICH Consolidated Guidelines on GCP (E6) and to local law or regulations. The investigator should ensure that all persons assisting with the trial are adequately informed about the protocol, any amendments to the protocol, the trial treatments, and their trial-related duties and functions. There are no plans for granting public access to the full protocol, participant-level dataset, or statistical code.

All information concerning the trial is confidential before publication. Interim data or final data can only be published in agreement between the coordinating investigator and Dr. F. Köhler Chemie GmbH. Publication of the results in an international peer-reviewed journal is planned at the end of study, in consultation and with the agreement of representatives of the centre.

## Discussion

Custodiol-N is based on the principles of the Custodiol® solution. Although there are no extensive study data available, we can refer to more than 30 years of experience with the use of Custodiol®. Since the pharmaceutical production of Custodiol® solution began, it is estimated to have been used in more than three million patients. The heart, the kidneys, the liver, the pancreas and even autologous vein grafts (in the context of coronary artery surgery) have all been treated with the solution. During the past 5 years in Germany alone, about 250,000 interventions in cardiac surgery were performed using Custodiol®.

The special value of Custodiol® for the preservation of the heart, liver and kidney intended for transplantation is emphasized by the fact that Custodiol® solution is regarded by Eurotransplant as one of the standards for preservation of these organs. Custodiol® solution is now in use in more than 90 countries worldwide for intraoperative myocardial protection and organ preservation.

Side effects specific to Custodiol® solution or induced by it have not been described in any part of the world, provided that the product had been used in accordance with its directions.

Based on experimental findings using traditional Custodiol® solution, a new Custodiol® solution (Custodiol-N) has been developed. Except for a few alterations, the successful composition of Custodiol® has been maintained with a high degree of safety, tolerability and efficacy. However, Custodiol-N proved to be superior to Custodiol® solution in in vitro studies concerning inhibition of hypoxic cell injury, inhibition of cold-induced cell injury and avoidance of adverse effects during warm exposure to the solution. The aim of this investigation is to demonstrate noninferiority of graft preservation with Custodiol-N compared to Custodiol® with respect to both graft function and injury after transplantation of kidney, liver or pancreas.

## Trial status

The study started in March 2019. The datasets analysed during the current study are available from the corresponding author on reasonable request. The overall duration for the trial is expected to be approximately 48 months. Protocol number of the study protocol: version 2.2. from 28 January 2019.

## Supplementary information

**Additional file 1.** SPIRIT 2013 checklist: recommended items to address in a clinical trial protocol and related documents.

## Data Availability

There are no data available yet; patient enrolment will start in March 2019.

## Abbreviations

AE: Adverse event
ALT: Alanine aminotransferase
ASA: American Society of Anesthesiologists
AST: Aspartate aminotransferase
AUC: Area under the curve
eCRF: Electronic case report form
GCP: Good clinical practice
HTK: Histidine-tryptophan-ketoglutarate
ICH: International Council for Harmonisation
LDH: Lactate dehydrogenase
MDRD: Modification of diet in renal disease
SAE: Serious adverse event
SUSAR: Suspected unexpected serious adverse reaction
